# Convolutional Neural Network-Based Human Detection in Nighttime Images Using Visible Light Camera Sensors

**DOI:** 10.3390/s17051065

**Published:** 2017-05-08

**Authors:** Jong Hyun Kim, Hyung Gil Hong, Kang Ryoung Park

**Affiliations:** Division of Electronics and Electrical Engineering, Dongguk University, 30 Pildong-ro 1-gil, Jung-gu, Seoul 100-715, Korea; zzingae@dongguk.edu (J.H.K.); hell@dongguk.edu (H.G.H.)

**Keywords:** intelligent surveillance system, nighttime human detection, visible light image, convolutional neural network

## Abstract

Because intelligent surveillance systems have recently undergone rapid growth, research on accurately detecting humans in videos captured at a long distance is growing in importance. The existing research using visible light cameras has mainly focused on methods of human detection for daytime hours when there is outside light, but human detection during nighttime hours when there is no outside light is difficult. Thus, methods that employ additional near-infrared (NIR) illuminators and NIR cameras or thermal cameras have been used. However, in the case of NIR illuminators, there are limitations in terms of the illumination angle and distance. There are also difficulties because the illuminator power must be adaptively adjusted depending on whether the object is close or far away. In the case of thermal cameras, their cost is still high, which makes it difficult to install and use them in a variety of places. Because of this, research has been conducted on nighttime human detection using visible light cameras, but this has focused on objects at a short distance in an indoor environment or the use of video-based methods to capture multiple images and process them, which causes problems related to the increase in the processing time. To resolve these problems, this paper presents a method that uses a single image captured at night on a visible light camera to detect humans in a variety of environments based on a convolutional neural network. Experimental results using a self-constructed Dongguk night-time human detection database (DNHD-DB1) and two open databases (Korea advanced institute of science and technology (KAIST) and computer vision center (CVC) databases), as well as high-accuracy human detection in a variety of environments, show that the method has excellent performance compared to existing methods.

## 1. Introduction

Intelligent surveillance is an important subject in the field of computer vision and is currently being actively researched [1,2,3,4,5,6,7,8,9,10,11]. Of particular importance is research on surveillance systems that operate well in the nighttime, when many crimes occur. With the development of hardware and algorithms, human detection for surveillance in complex environments has recently become possible, and in particular, the accuracy of human detection using convolution neural networks (CNNs) has greatly improved [12,13]. However, human detection at night using visible light cameras remains a difficult problem. This is because the features needed for human detection sometimes do not exist, to the point where it is difficult to find human areas even with the human eye, and there is an outstanding amount of noise observable. Thus, there has been a large amount of research performed on human detection using far-infrared (FIR) cameras instead of the widely used visible light cameras [14,15,16,17,18,19,20,21,22,23,24,25,26]. Moreover, research has been performed on human detection by fusing visible light cameras and IR cameras to more accurately obtain features from nighttime images [27]. In addition, research has been performed on human detection at night using only visible light cameras [28,29]. Section 2 examines these research efforts and points out their pros and cons.

## 2. Related Works

The existing research on human detection at night can be broadly divided into multiple camera-based methods and single camera-based methods. Multiple camera-based methods work by separately extracting features from videos captured by infrared (IR) cameras and visible light cameras and fusing them together. However, because there are usually almost no features seen by the visible light cameras at night, the emphasis is placed on the IR cameras. For example, the research in [27] used continuous nighttime FIR video frames and visible video frames. First, spatial-temporal filtering was used on the FIR video frames to remove noise. Because the human body usually has a higher temperature than the background, human candidate pixels were created with the assumption that they should have a high intensity in the IR image. However, because a pixel can have high intensity due to noise, temporal information was used to designate human pixels only when they had a high intensity for more than a fixed time. After this, a seeded region growing method was used on the human pixels to segment out a human area. The segmented area’s aspect ratio was used to perform verification and remove inaccurately detected human areas. Finally, a min-max score fusion was used to perform fusion with the visible video frames. Although the research in [27] used the assumption that the human body has a higher temperature than the background, during a hot summer, the background can be hotter than or similar to the human body, even at night. In addition, because it used the video frame-based spatial-temporal filtering method to remove noise, it had the limitation of not being usable for a single image. In addition, calibration is usually required for a multiple camera-based method to fuse the visible light camera and IR camera, and it is an inconvenience to have to manually set corresponding points between the two cameras. Beyond this, there are other drawbacks in that FIR camera prices are high (more than $7500 for 640 × 480 pixels devices [30]). Thus, they have limited applicability to ordinary surveillance environments. The two cameras' videos must be processed, which takes a long time, and the processing speed is lowered when a large number of objects is detected.

Because of these problems, single camera-based methods are being researched. In the case of single camera-based methods, there is research on using only IR cameras to perform human detection [14,15,16,31]. In [14], a Gaussian mixture model (GMM) was used on FIR image frames to find the background intensity distribution of each pixel. Then, when a new FIR image appeared, the probability of the background was calculated based on the background intensity distribution, and if this probability was low, it was determined to be the foreground and human detection was performed. In this approach, only a fixed camera could be used because the intensity distribution of each pixel’s background had to be obtained. In the research of [15] as well, humans were assumed to have a higher temperature than the background, and a human’s size was estimated based on regions with high intensity. The pixels of the estimated regions of interest (ROIs) were made into feature vectors, and human classification was performed via a support vector machine (SVM). In the research of [16], histograms of oriented gradients (HOGs) from the ROIs in the video captured from near-infrared (NIR) or FIR cameras were used to create feature vectors, and human classification was performed via the SVM. In previous research [31], a monocular vision system for real-time pedestrian detection and tracking was proposed for nighttime driving with a NIR camera with illumination from full-beam headlights. Their method was composed of three parts in a cascade such as ROI generation based on the dual-threshold segmentation, object classification based on a tree-structured two-stage detector, and template-matching-based tracking. In addition, each part uses complementary visual features to discriminate the objects from the cluttered background in the range of 20–80 m. However, in the methods used in [15,16], thresholding methods were used to find human candidate regions. Thus, it was possible for human areas not to be recognized as candidates for classification as a result of thresholding errors. In addition, usually when an NIR camera is used at night, an additional illuminator must be used, and the illumination has angle and distance limitations. Although the power of full-beam headlights is sufficient to detect pedestrian in the range of 20–80 m, the illuminator’s power must also be adaptively adjusted in order to prevent the bright saturation of object in the captured image when objects are closer than 20 m [31]. In addition, FIR cameras, which are thermal cameras, have a high cost, which makes them difficult to use in a variety of environments. FIR cameras also usually have an image resolution that is far below that of visible light cameras. Therefore, they have the disadvantage of being able to capture few features in a human region during human detection from a long distance.

For these reasons, research is being performed on human detection at night using visible light images [28,29]. Such research uses continuous visible light video frames to perform pedestrian detection. That is, pedestrian detection is performed based on the contrast information and frame-to-frame contrast changes in a single image. This method has limitations in that it is difficult to detect a human who is standing very still, a fast video capture speed is needed because continuous video frames must be used, and multiple frames must be processed, which increases the processing time.

Normally, finding features for human detection without an image enhancement process is difficult when using visible light images under low illumination conditions such as nighttime. Thus, a variety of image enhancement methods have been studied [32,33,34,35,36,37,38,39]. Traditional methods include histogram equalization (HE) and histogram specification (HS) [32]. However, if these methods are used, the noise is also increased and important low frequency components are decreased. To resolve these problems, research has been performed on a variety of histogram processing and intensity mapping-based image enhancement methods [33,34,35,36,37]. There has also been research on different methods that perform denoising followed by image enhancement [38,39]. However, because the denoising part involves many operations, it requires a longer processing time than histogram methods. Additionally, the above methods [32,33,34,35,36,37,38,39] have only demonstrated experimental results in raising the image visibility through image enhancement, but have not shown results in human detection in nighttime images, which was the goal of this study.

Although they have not researched human detection, as the category of feature detection and analysis of object, they proposed the method for facial landmark localization and descriptors for face analysis [40,41]. In [40], they proposed the method of point-by-point mapping, of 11 differential geometry descriptors such as curvatures to the three individual RGB image components. From that, three-dimensional features are used for representing bidimensional facial image to extract the facial landmark positions. In [41], they proposed 105 geometrical descriptors for face analysis. The descriptors were generated by composing primary geometrical descriptors such as principal curvatures, Gaussian, mean, shape index, curvedness, and the coefficients of the fundamental forms, and by applying conventional functions such as cosine, sine, and logarithm to them. These descriptors were projected on 217 facial depth maps and analysed in terms of exploitability for localizing landmark points and descriptiveness of facial shape. Although their methods showed the high performance in facial landmark localization and as descriptors for face analysis, it is difficult to apply these methods for human detection in a single nighttime image at a distance.

As the feature extractor, the HOG method has been widely applied to various computer vision problems using face images or human body, such as the pedestrian detection [16,42], age estimation [43], face recognition [44], and gender recognition [45,46]. The HOG method constructs histogram features of a sub-block of an image based on the accumulation of the direction and strength of the gradient information at every pixel within the sub-block.

Considering the problems and limitations of the existing research, this research investigated a method for human detection in a single nighttime image captured by a visible light camera using a CNN. We also compared the performances using the original images and HE images as the CNN input. In addition, we compared the accuracy by our method with that by the HOG-based method. Our research was novel compared to previous works in the following four ways:

1. This was the first research on human detection using a single visible light camera image in an outdoor long-distance nighttime low-illumination environment.
2. We performed intensive training on the CNN using a huge number of images obtained through data augmentation from three kinds of nighttime databases of images captured in a variety of environments by fixed and moving cameras in order to improve the CNN-based human detection performance, making it robust for a variety of cameras and environment changes.
3. We compared the performance of an original image-based CNN and an HE image-based CNN in order to compare the properties and human detection performances based on the relationship between the nighttime image capturing environment and the image pre-processing. In the analysis results, when HE images were used rather than the original images and the three databases were combined for training rather than training being done separately for each database, the system showed better human detection performance.
4. The test database was self-constructed using images obtained from cameras installed in nighttime surveillance environments, and this database has been made public so that other researchers can compare and evaluate its performance.

Table 1 lists the results of a comparison of the proposed method and those from previous studies on human detection in nighttime images.

The remainder of this paper is organized as follows: Section 3 describes the proposed system and method of human detection. In Section 4, the experimental setup, results, and analyses are shown. Finally, the conclusions are presented in Section 5.

## 3. Proposed Human Detection in Nighttime Image Based on CNN

### 3.1. Overall Procedure of Proposed Method

**Figure 1:**
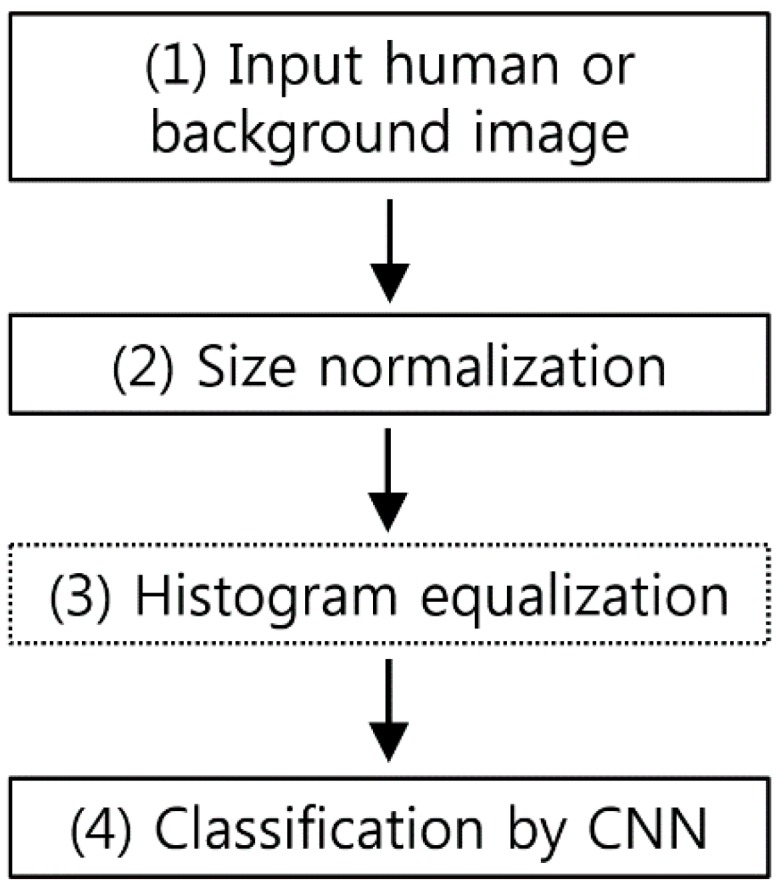
Flowchart of proposed method.

Figure 1 is a flowchart of the CNN-based human detection method presented in this paper. Because the size of the input human or background image varies depending on the image, size normalization is performed (step (2) of Figure 1) via bilinear interpolation in order to obtain an image with a fixed size (height of 183 pixels and width of 119 pixels). AlexNet [47] and other existing methods from the literature [48,49] used square input images with the same height and width. However, for a normal human area, which is the target of this research, the height is far greater than the width. Thus, when square-shape size normalization is performed, the image is stretched too much in terms of the height vs. width, which distorts the human area and makes accurate feature detection difficult. Moreover, if the CNN input images are square, with no horizontal stretching, too much image background area to the left and right of the person is included, in addition to the human area, which results in inaccurate feature detection. To take this into account, this research used 183 × 119 pixel (height × width) normalized human or background images as the CNN input. Through this size normalization, we compensated for size changes in the objects when they were close to or far away from the camera. In addition, in this research, we used the zero-center method to perform brightness normalization on the input images [50]. An input image of 183 × 119 pixels (height × width) is much smaller than the 227 × 227 pixel (height × width) images used in AlexNet [47]. Therefore, the number of filters in each convolutional layer and the number of nodes in fully connected layers can be smaller than those in AlexNet. In addition, AlexNet was designed to classify 1000 classes, which requires a more complex structure. In contrast, in this research, the human and background areas were divided into only two classes, which reduced the training time. After size normalization, we compared the performances when HE was performed to adjust the brightness and when HE was not performed (see the details in Section 4). Then, the image was used as the input for the trained CNN and classified as either a human or background area (step (4) of Figure 1).

### 3.2. Convolutional Layers of CNN

**Figure 2:**
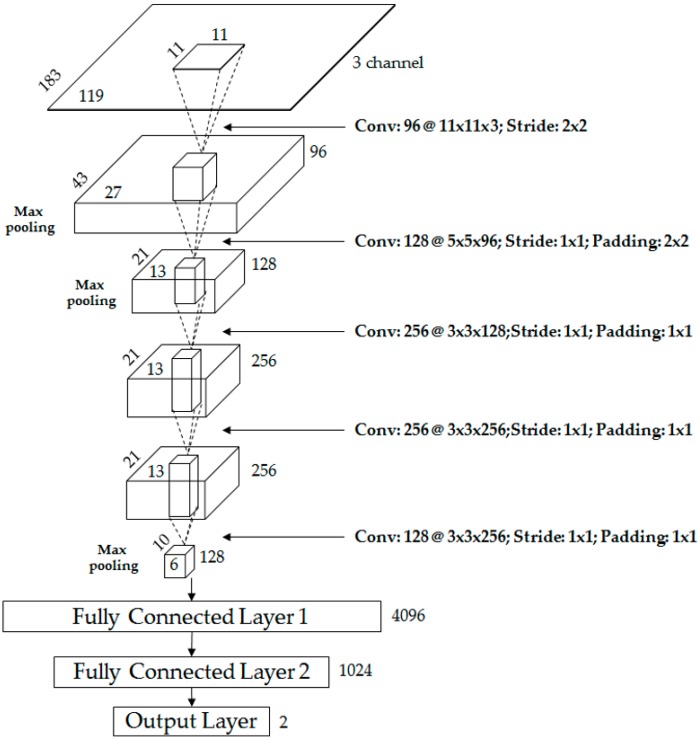
Proposed CNN architecture.

Table 2 and Figure 2 show the CNN structure used in this research. It is composed of five convolutional layers and three fully connected layers. In the 1st convolutional layer, 96 filters with a size of 11 × 11 × 3 are used at a stride of 2 × 2 pixels in the horizontal and vertical directions.

The size of the feature map is 87 × 55 × 96 in the 1st convolutional layer, with 87 and 55 as the output height and width, respectively, calculated as follows: (output height (or width) = (input height (or width) − filter height (or width) + 2 × padding)/stride + 1 [51]). For example, in Table 2, the input height, filter height, padding, and stride are 183, 11, 0, and 2, respectively, and the final output height is 87 (=(183 − 11 + 2 × 0)/2 + 1). The 1st convolutional layer’s filter size is relatively large, unlike those in previous studies [52,53], because the input images are dark, with a fairly large amount of noise in their features. Therefore, the filter size was increased to prevent invalid features from being produced as a result of noise. The rectified linear unit (ReLU) layer was used in the form shown in Equation (1) [54,55,56].

(1) y=max(0,x)

where *x* and *y* are the input and output values, respectively. This function can reduce the vanishing gradient problem [57] that might occur when a sigmoid or hyperbolic tangent function is adopted in back-propagation for training and has a faster processing speed than a non-linear activation function.

After the ReLU layer, we used a cross channel normalization layer, as shown in Table 2, which has the following formula:

(2) $$c_{x,y}^{i}=\frac{b_{x,y}^{i}}{{\left(p+\alpha \sum_{j=max\left(0,i-\frac{n}{2}\right)}^{min\left(N-1,i+\frac{n}{2}\right)}{\left(a_{x,y}^{j}\right)}^{2}\right)}^{\beta }}$$

In Equation (2), $c_{x,y}^{i}$ is a value obtained by normalization [47]. In this research, we used 1, 0.0001, and 0.75 for the values of *p*, *α*, and *β*, respectively. $b_{x,y}^{i}$ is the neuron activity computed by the application of the ith kernel at location (*x*, *y*), and it performs normalization for the adjacent n kernel maps at the identical spatial position [47]. In this research, *n* was set at 5. N represents the total number of kernels in the layer.

To make the CNN structure robust with regard to image translation and local noise, the feature map obtained after passing through the cross channel normalization layer was passed through the max pooling layer, as shown in Table 2. The goal was to perform a kind of subsampling by selecting the maximum value from the values included in the mask range determined in the max pooling layer and using this as the final value. After passing through the max pooling layer, we obtained the 96 feature maps with a size of 43 × 27 pixels, as shown in Table 2 and Figure 2.

After using the 1st convolutional layer, we used the 2nd convolutional layer, which had 128 filters with a size of 5 × 5 × 96, stride of 1 × 1 pixels (in the horizontal and vertical directions), and padding of 2 × 2 pixels (in the horizontal and vertical directions), as shown in Table 2 and Figure 2. After the 1st convolutional layer, we used the ReLU, cross channel normalization, and max pooling layers and obtained 128 feature maps with a size of 21 × 13 pixels, as shown in Figure 2 and Table 2. The first two layers were used to extract low-level image features such as blob texture features or edges. Afterwards, an additional three convolutional layers were used for high-level feature extraction, as shown in Figure 2 and Table 2. In detail, the 3rd convolutional layer adopted 256 filters with the size of 3 × 3 × 128, the 4th convolutional layer had 256 filters with a size of 3 × 3 × 256, and the 5th convolutional layer adopted 128 filters with a size of 3 × 3 × 256.

### 3.3. Fully Connected Layers of CNN

Through these five convolutional layers, 128 feature maps with a size of 10 × 6 pixels were finally obtained. These were fed to an additional three fully connected layers, which included 4096, 1024, and two neurons, respectively. In this research, the human (foreground) area and background area were ultimately classified into two classes via the CNN, and the last (3rd) fully connected layer (called the “output layer”) from Figure 2 and Table 2 only had two nodes. In the 3rd fully connected layer, the softmax function was used, as shown in Equation (3) below [55].

(3) $$\sigma {\left(s\right)}_{j}=\frac{e^{sj}}{\sum_{n=1}^{K}e^{sn}}$$

Given that the array of output neurons was set at s, we could obtain the probability of neurons belonging to the *j*^th^ class by dividing the value of the *j*^th^ element by the summation of the values of all the elements. As described in previous studies [47,58], CNN-based recognition systems have an over-fitting problem, which can cause a low recognition accuracy with testing data, even though the accuracy with the training data is still high. To resolve this problem, in this research we used data augmentation and dropout methods [47,58], which could reduce the effects of the over-fitting problem. For a detailed description of the experiment data created through data augmentation, see Section 4.1. For the dropout method, we adopted a dropout probability of 50% to disconnect the connections for several neurons between the previous layer and next layers in the fully connected network [47,58]. The dropout layer was only used directly before the 3rd fully connected layer, as shown in Table 2.

## 4. Experimental Results

### 4.1. Experimental Data and Environment

**Figure 3:**
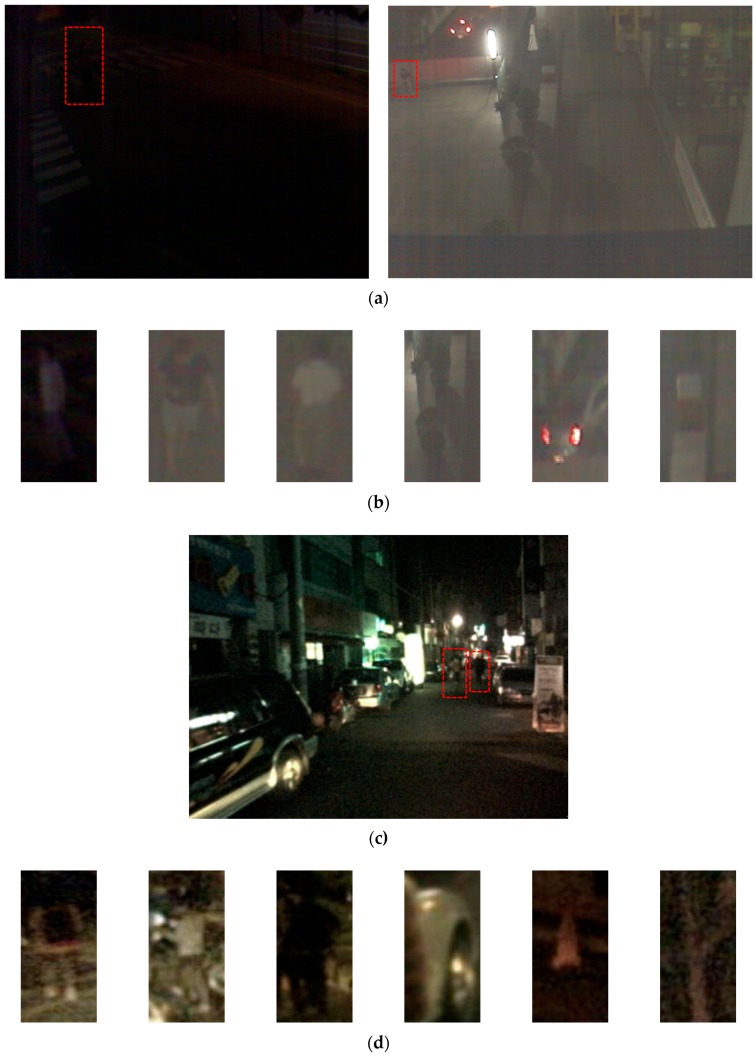

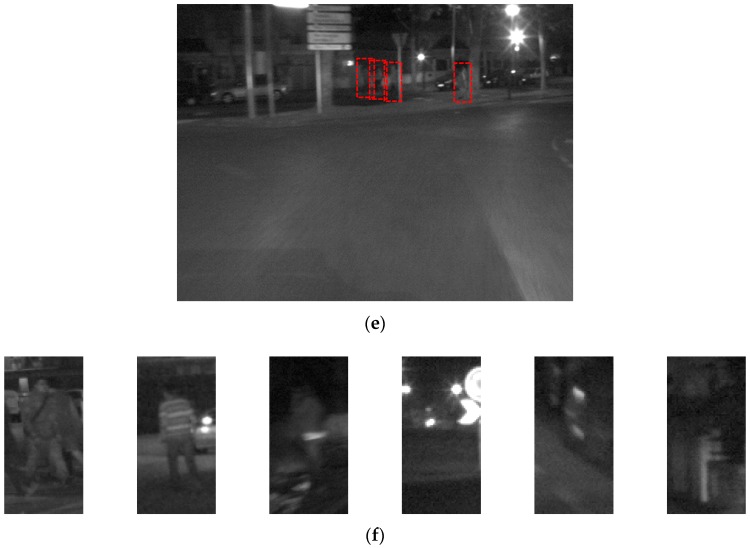
Examples from three databases used in experiments: (**a**) collected images of DNHD-DB1; (**b**) human and background images of DNHD-DB1; (**c**) collected image of KAIST database; (**d**) human and background images of KAIST database; (**e**) collected image of CVC-14 database; and (**f**) human and background images of CVC-14 database. In (**a**,**c**,**e**), human areas are shown as red dashed box. In (**b**,**d**,**f**), left three figures are human images whereas the right three ones are background images, respectively.

In this research, three databases were used for a performance evaluation. The first database was the self-constructed Dongguk nighttime human detection database (DNHD-DB1) as shown in Figure 3a,b [59], which was obtained through a nighttime visible light camera (Logitech C600 web camera [60]). DNHD-DB1 was constructed using images obtained from cameras fixed at various locations, and it has been made public through [59] so that outside researchers can use it. In addition, in order to evaluate the human detection performance in nighttime images captured with a moving camera rather than a fixed camera, we used the KAIST [61] and CVC-14 databases [62] as shown in Figure 3c–f in the experiments. These two databases include images of pedestrians on the street, which were obtained using cameras attached to cars.

To perform four-fold cross validation training and testing, each of the databases was divided into four subsets. At this point, each subset had an almost equal ratio of human and background images, and the images in each subset were chosen through random selection.

As described in Section 3.3, data augmentation was performed based on the database’s training data in order to resolve the over-fitting problem. The data augmentation method used in this research can be described as follows. Images were translated and cropped by 1 pixels at a time in the top, bottom, left, and right directions based on the coordinates for the original human and background images, and these images were all saved so that 24 augmented images were obtained from a single original image. An additional image was also obtained from a single original image using horizontal reflection. Thus, a total of 25 augmented images were obtained from a single original image, and the original image and augmented images were used in training. This kind of data augmentation was used because the number of original human images was small. However, in the case of background images, we were able to select a large number of images with non-human areas from the input images. Thus, we did not use data augmentation but randomly selected the parts without humans to increase the amount of data. For the CNN training and testing, we used a desktop computer with an Intel^®^ Core™ i7-6700 CPU @ 3.40 GHz (Intel Corporation, Santa Clara, CA, USA) (4 CPUs) [63], 64 GB memory, and a NVIDIA GeForce GTX TITAN X (3072 CUDA cores) (Nvidia Corporation, Santa Clara, CA, USA) graphics card with 12 GB memory [64]. The CNN algorithm was created in MATLAB (The MathWorks, Inc., Natick, MA, USA) [50].

### 4.2. Training of CNN Model

In this research, we trained the CNN model in two ways. The first was to train it with the augmented data obtained from the three databases, using each database independently (separate database), and the second was to train it with the augmented data obtained from the three databases, using a combined database (combined database). As listed in Table 3, the images in the CVC-14 database are 1 channel gray scale images, unlike the images in the other databases. Thus, the experiments were performed with databases that included images where the RGB channels for each pixel had the same gray intensity value.

Here, as described in Figure 1, the training images were divided into the original images with no brightness correction and HE enhanced images. The training was performed, and the performance was evaluated using four-fold cross validation. The augmented data were only used in the training process, with only the non-augmented original images used in the testing process for the performance evaluation.

A total of 24 (2 × 3 × 4) independent CNN weights were trained with four-fold cross validation using the original images and HE images from the three databases (separate databases). In addition, eight (2 × 4) independent CNN weights were trained with four-fold cross validation using the original images and HE images from the combined database. Ultimately, in this experiment, 32 (24 + 8) CNN weights were trained, and the testing performances for the 32 were each evaluated.

In this research, we used the stochastic gradient descent (SGD) method [65] for the CNN training. The SGD method finds the derivative of the optimal weight that minimizes the difference between the desired output and the calculated output. Unlike the gradient descent (GD) method, in the SGD method, the number of mini-batch size units that the training set is divided into is defined as an iteration. When training has been performed for the total number of iterations, this is called an epoch, and the training is performed for a preset number of epochs. The parameters used for the SGD method in this study were as follows: InitialLearnRate = 0.001, L2Regularization = 0.0001, MiniBatchSize = 128, and Momentum = 0.9. The meaning of each parameter can be found in [66]. In the first training, the sample data were shuffled. The weights used in this research were initialized randomly using a Gaussian distribution with mean = 0 and standard deviation = 0.01, and the biases were initialized at a default value of zero.

**Figure 4:**
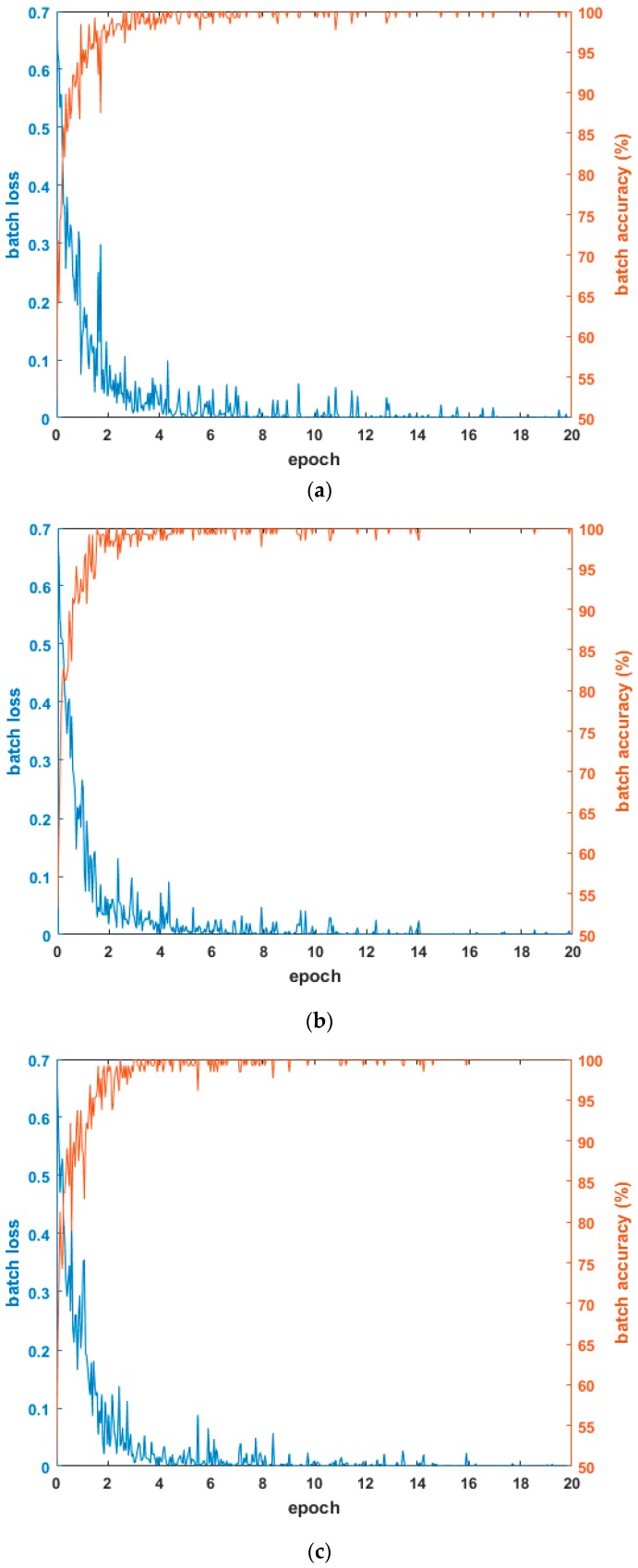

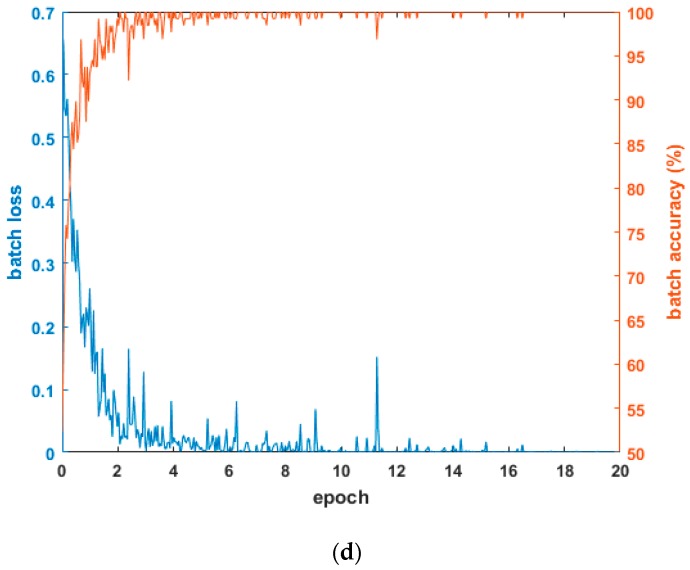
Examples of loss and accuracy curves with training data of four-fold cross validation in case of using combined database images: (**a**) 1st fold; (**b**) 2nd fold; (**c**) 3rd fold; and (**d**) 4th fold.

Figure 4 shows the graphs of loss and accuracy according to the epoch number in the training procedure. As shown in the tables in Section 4.3.1 and Section 4.3.2, the testing accuracies when using the combined database of HE processed images were higher than other cases. Therefore, Figure 4 shows the loss and accuracy curves with the four-fold cross validation training data when using the combined database of HE processed images. As shown in Figure 4, in all cases, as the epoch number increased, the loss curve approached zero, and the training accuracy approached one (100%). Based on this, we know that the CNN training was performed successfully.

**Figure 5:**
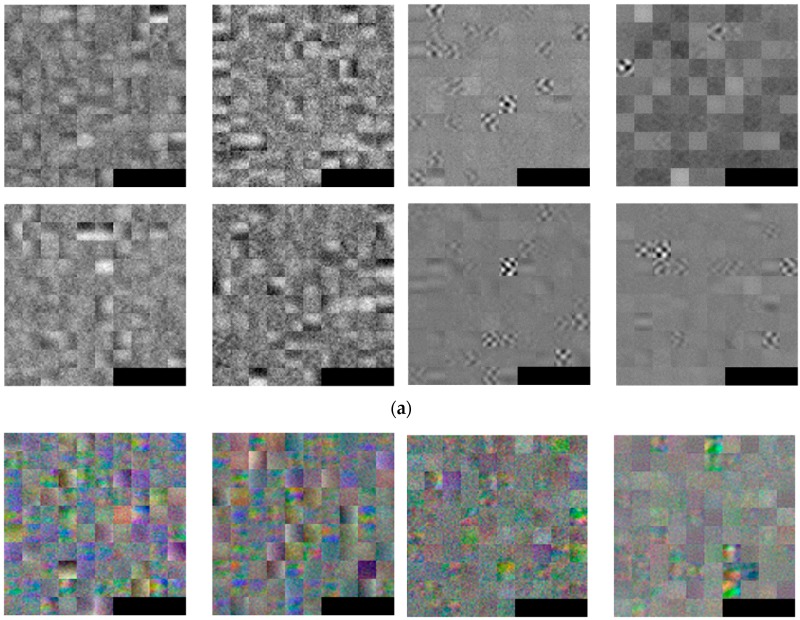

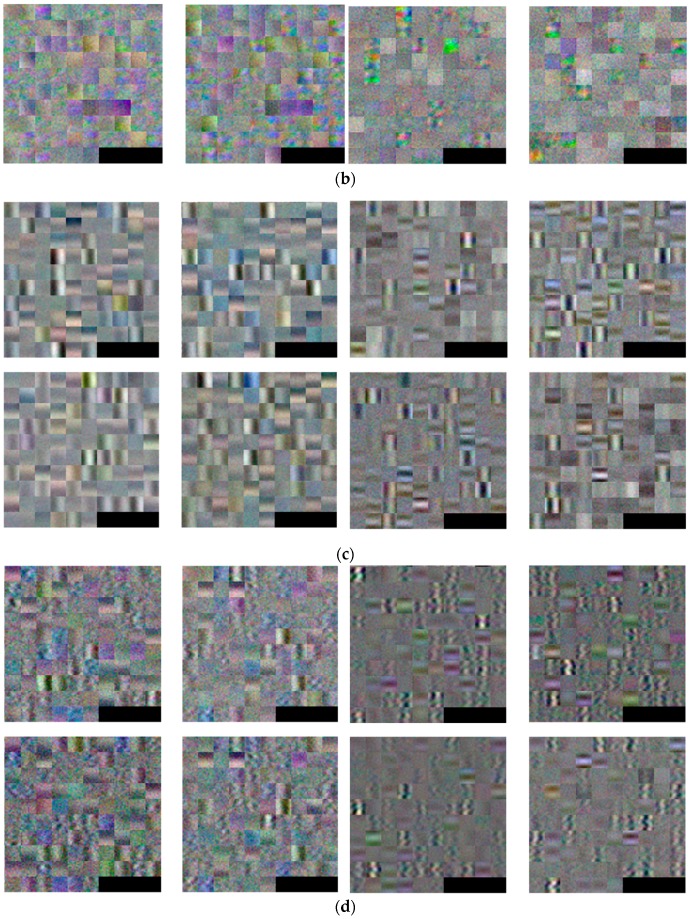
Examples of 96 filters obtained from 1st convolution layer through training: (**a**) CVC-14 database; (**b**) DNHD-DB1; (**c**) KAIST database; and (**d**) combined database. In (**a**–**d**), the left four images show the 96 filters obtained by training with the original images, whereas the right four images represent those obtained by training with the HE images. In the left and right four images, the left-upper, right-upper, left-lower, and right lower images show the 96 filters obtained by training using 1-, 2-, 3-, and 4-fold cross validation, respectively.

Figure 5 shows an example of the 96 filters ((11 × 11 × 3) as listed in Table 2) in the 1st convolutional layer obtained through this training process. For visibility, the 11 × 11 filters are visualized at three times their size. The filters obtained with the CVC-14 database are gray images, as shown in Figure 5a, because the images in the CVC-14 database are gray (1 channel), as listed in Table 3. All of the other filters are color images, as shown in Figure 5b–d.

Looking at the 96 filters obtained from the original images in the KAIST database on the left side of Figure 5c, we can see that there is relatively little noise, and there is a marked contrast between the left and right or up and down directions in the filters compared to the 96 filters obtained from the original images of the CVC-14 and DNHD-DB1 databases in Figure 5a,b, respectively. From this we know that the KAIST database’s original images have a lower noise level than those in the original images of the CVC-14 and DNHD-DB1 databases, and there are relatively large differences between the people/background areas and their surroundings.

### 4.3. Testing of Proposed CNN-Based Human Detection

#### 4.3.1. Testing with Separate Database

As explained in Section 4.3, the trained CNN was used to evaluate the testing performances with the three databases individually (separate databases). For the testing performance evaluation, the human (foreground) area and background area were defined as positive and negative data, respectively, and from this we defined true negatives (TNs), true positives (TPs), false negatives (FNs), and false positives (FPs). A TN was a case where the background area was correctly recognized as a background region, whereas a TP was a case where the human area was correctly recognized as a human region. A FN was a case where the human area was incorrectly recognized as a background region, whereas a FP was a case where the background area was incorrectly recognized as a human region. Based on these, we could define errors using the false negative rate (FNR) and false positive rate (FPR). In addition, two accuracy measurements could be defined using the true positive rate (TPR) and true negative rate (TNR). The TPR was calculated as 100 − FNR (%), and the TNR was calculated as 100 − FPR (%).

Table 4 and Table 5 list the TPR, TNR, FNR, and FPR as confusion matrices. For example, in Table 4a, the TPR, TNR, FNR, and FPR are 96.59%, 99.73%, 3.41%, and 0.27%, respectively. In all the databases, the TPR and TNR had high values, and overall there was higher accuracy when using the HE processed images than when using the original images.

**Figure 6:**
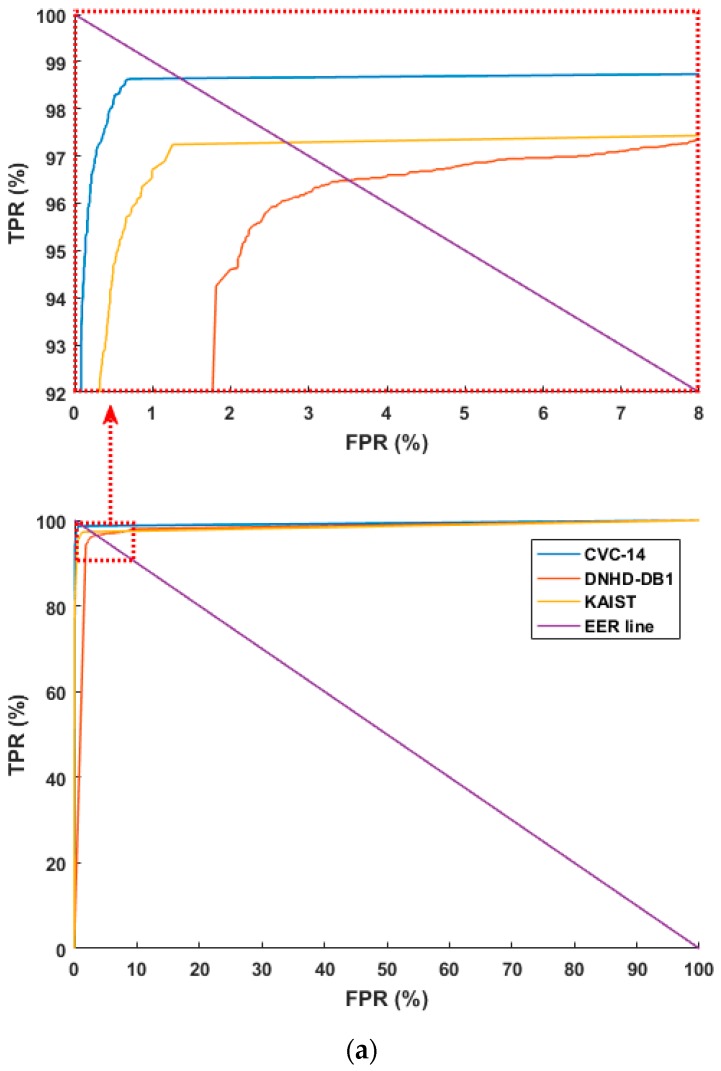

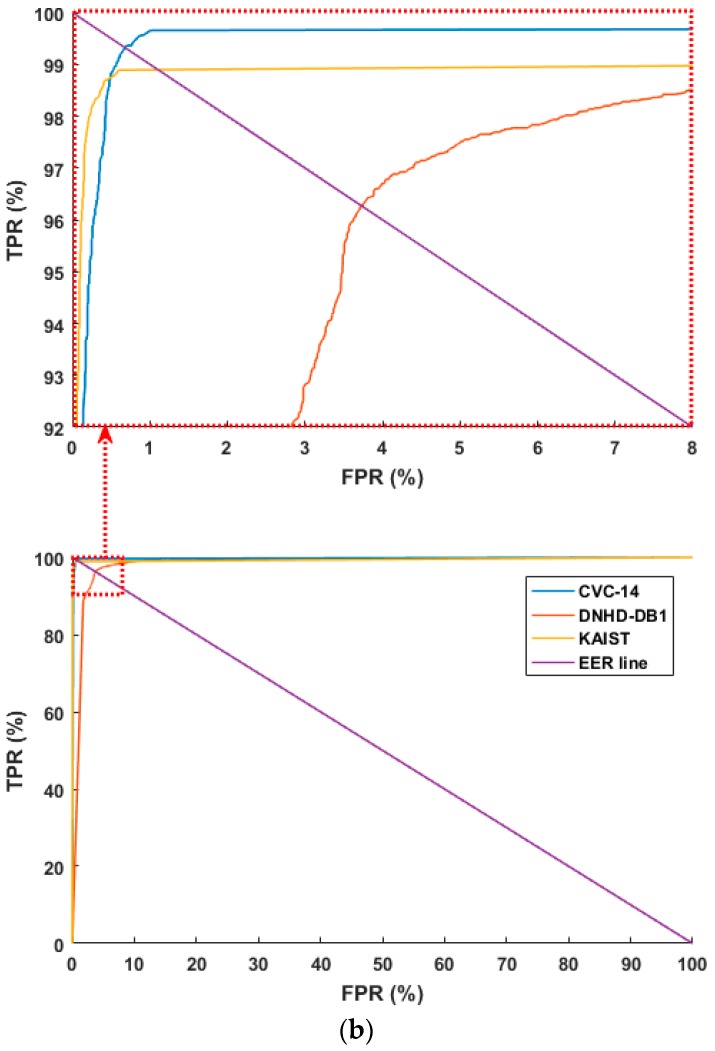
ROC curves of human and background detection: (**a**) with original images, ROC curves in the part ranges of FPR and TPR (**top**), ROC curves in the whole ranges of FPR and TPR (**bottom**) and (**b**) with HE processed images, ROC curves in the part ranges of FPR and TPR (**top**), ROC curves in the whole ranges of FPR and TPR (**bottom**).

Figure 6 shows the receiver operating characteristic (ROC) curves based on the aforementioned FPR and TPR (100 – FNR (%)). Figure 6 shows the average of the four curves obtained through four-fold cross validation. As shown in Figure 6, the accuracies with the CVC-14 database are higher than those with the other databases in both cases of using original images and HE processed ones. In addition, based on the aforementioned TP, TN, FP, and FN, we used the following four criteria for accuracy measurements [67]:

(4) $$\mathrm{Positive}\;\mathrm{predictive}\;\mathrm{value}\;\left(\mathrm{PPV}\right)=\frac{\#\mathrm{TP}}{\#\mathrm{TP}+\#\mathrm{FP}}$$

(5) $$\mathrm{TPR}=\frac{\#\mathrm{TP}}{\#\mathrm{TP}+\#\mathrm{FN}}$$

(6) $$\mathrm{Accuracy}\;\left(\mathrm{ACC}\right)=\frac{\#\mathrm{TP}+\#\mathrm{TN}}{\#\mathrm{TP}+\#\mathrm{TN}+\#\mathrm{FP}+\#\mathrm{FN}}$$

(7) $$F\_\mathrm{score}=2\cdot \frac{\mathrm{PPV}\cdot \mathrm{TPR}}{\mathrm{PPV}+\mathrm{TPR}}$$

where #TP, #TN, #FP, and #FN mean the numbers of TP, TN, FP, and FN, respectively. The minimum and maximum values of PPV, TPR, ACC, and F_score are 0 (%) and 100 (%), respectively, where 0 (%) and 100 (%) represent the lowest and highest accuracies, respectively. Based on these, we list the accuracies in Table 6 and Table 7. In these tables, the accuracies are the averages of the four testing accuracies obtained through four-fold cross validation.

**Figure 7:**
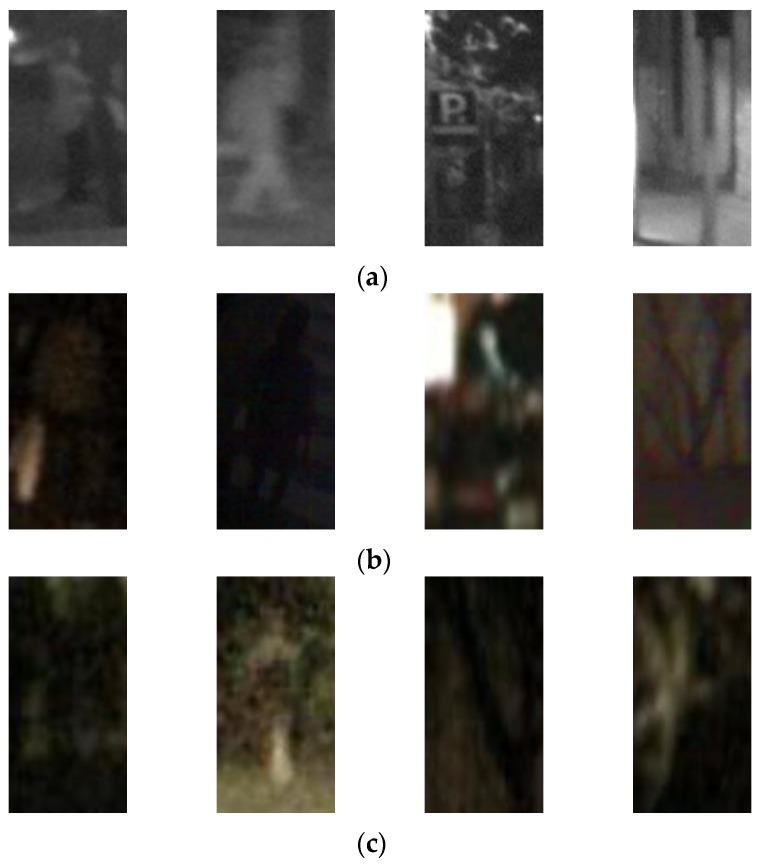
Examples of FN and FP errors in cases using original images of (**a**) CVC-14 database; (**b**) DNHD-DB1; and (**c**) KAIST database. In (**a**–**c**), the left two images show the FN cases, whereas the right two images represent the FP cases.

**Figure 8:**
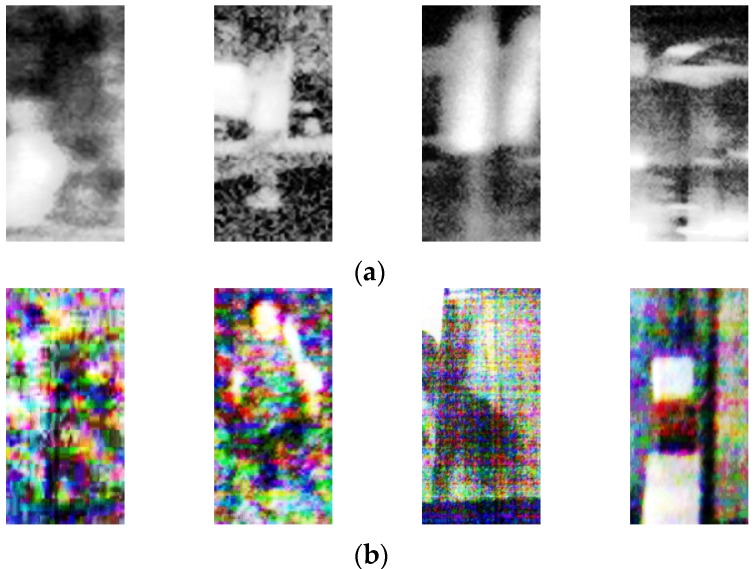

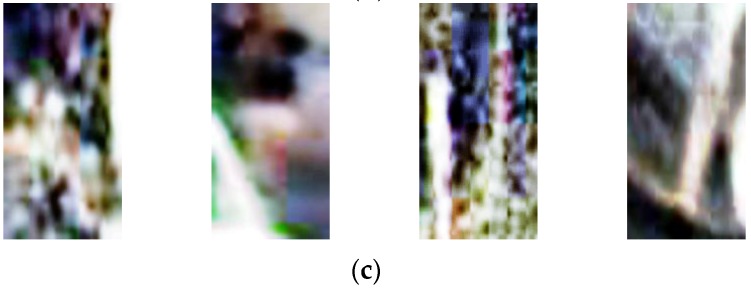
Examples of FN and FP errors in cases using HE processed images of (**a**) CVC-14 database; (**b**) DNHD-DB1; and (**c**) KAIST database. In (**a**–**c**), the left two images show the FN cases, whereas the right two images represent the FP cases.

By comparing the accuracies listed in Table 6 and Table 7, we can find that the average accuracies with the images processed by HE are higher than those with the original images. Figure 7 and Figure 8 show examples of the FP and FN errors. As shown in Figure 7, when a human’s approximate silhouette is observed in a dark environment, FP errors appear in many cases where the background’s silhouette is almost the same as the human because it is dark, and the two cannot be distinguished, while FN errors involve cases where the image is so dark that it is almost impossible to observe human silhouette information even using the human eye.

As shown in Figure 8, the FP and FN errors occur when the human and background images look similar even with HE processing. In addition, when HE is applied to dark image, the noise level is also increased with the image contrast, which causes the FP and FN errors.

#### 4.3.2. Testing with Combined Database

The next experiment used a combined database made from a combination of the three databases (CVC-14 database, DNHD-DB1, and KAIST database) to estimate the PPV, TPR, ACC, and F score through four-fold cross validation, and the results are listed in Table 8. In the experimental results, the testing accuracies for training after HE processing were higher than for training with the original images, and these numbers were higher than the results for the separate databases listed in Table 6 and Table 7. In all the experiments, using the combined database after HE processing produced the highest testing accuracy.

#### 4.3.3. Comparison with Previous Methods

**Figure 9:**
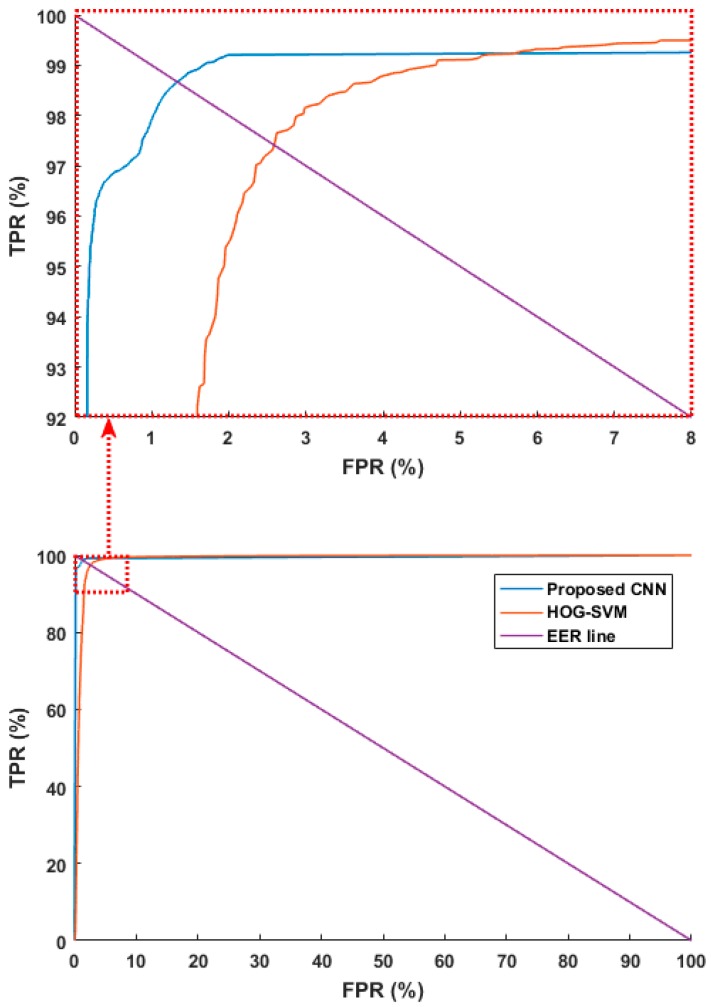
ROC curves of human and background detection by proposed and previous methods [16,42]. ROC curves in the part ranges of FPR and TPR (**top**), ROC curves in the whole ranges of FPR and TPR (**bottom**).

In another experiment, we compared the performance of the proposed CNN method with previous research [16,42] where image features were found using HOGs, and the humans and background were differentiated via the SVM. As in the previous experiment scheme, we used the combined database and tested it with four-fold cross validation. To make a fair comparison, we used the same augmented data used for training in the previous methods. Figure 9 shows ROC curves based on the aforementioned FPR and TPR. The figure shows the average of the four curves obtained through four-fold cross validation. As shown in Figure 9, the accuracies with the proposed method are higher than those using previous methods [16,42]. Next, the PPV, TPR, ACC, and F score were estimated as listed in Table 9. Like Table 8, it shows the average of the four accuracies obtained through four-fold cross validation. The experimental results showed that the proposed CNN method had higher accuracy than the existing methods.

## 5. Conclusions

In this research, we studied a CNN-based method for human detection in a variety of environments using a single image captured from a visible light camera at night. We used three databases of images with a variety of properties captured from both fixed and moving cameras to train the system to be robust through multiple environmental changes. When we compared the detection accuracies of a CNN using pre-processing by HE and a CNN that did not use this pre-processing, we found that the accuracy was higher when using a combined database with HE pre-processing. We also found that when the three databases were combined for training (combined database), the system showed higher human detection performance than when the training was performed with the three databases individually (separate databases). In addition, we created a self-constructed database for experiments based on cameras installed in nighttime surveillance environments at various locations and made this database public to allow other researchers to make performance comparisons. In the results we obtained, FP and FN errors only occurred in images captured in environments so dark that it was even difficult to recognize human silhouettes with the human eye.

Although our CNN-based method shows high performance in human detection in a nighttime image, the CNN network is to be trained and the training process for any deep net can be always its Achilles’ heel unless the trained net is provided for other users. Considering this issue, we made our trained net open to other researchers through [59], and would intensively research this issue in future work. In addition, we hope to use the CNN method proposed in this research to detect a variety of objects (e.g., cars, motorcycles, dogs, cats, etc.) other than humans at night. We also intend to conduct research on detection using low-resolution human images captured at much further distances than a normal surveillance environment for the purpose of military application.

## Figures and Tables

**Table 1:** Comparison of proposed method and previous study results.

Category	Method		Advantages	Disadvantages
Multiple camera-based method	Using visible light and FIR cameras [27]	Spatial-temporal filtering, seeded region growing, and min-max score fusion	Uses data from two cameras to improve human detection accuracy	- Correspondence points must be manually set between two cameras for calibration - Requires a sequence of image frames - Difficult to use in most normal surveillance environments because of high cost of FIR cameras - Images from two cameras must be processed, which takes a long time, and the processing speed is lowered if many objects are detected
Single camera-based methods	Using IR camera (NIR or FIR camera)	GMM [14], SVM classifier with feature vector from human region [15] and by HOG [16]	Uses one camera, which eliminates the need for calibration, and has a faster processing time than multiple camera-based methods	- Can only be used in a fixed camera environment [14] - If an NIR camera is used, an additional NIR illuminator must be used. NIR illuminators are limited in terms of their illumination angle and distance, and the illuminator power must be adaptively adjusted for near and far objects [16] - FIR cameras are expensive, and their image resolution is much lower than visible light cameras. Thus, there are few features that can be captured in a human area during human detection at a long distance [14,15,16]
	Using visible light camera	Uses local change in contrast over time [28,29]	Uses low-cost visible light cameras	- Can only be used in a fixed camera environment - Has trouble detecting humans who are standing still - Must use continuous video frames, which requires a fast capture speed, and it processes multiple images, which increases the processing time
		Histogram processing or intensity mapping-based image enhancement [32,33,34,35,36,37]	Uses low-cost visible light cameras	- Has only produced experimental results for raising the visibility through image enhancement, with no results produced for human detection in nighttime images
		Denoising and image enhancement [38,39]	Effectively removes noise that occurs during image enhancement	- Because denoising requires many operations, the processing time is long compared to histogram methods - Has only produced experimental results for raising visibility through image enhancement, with no results produced for human detection in nighttime images
		CNN (proposed method)	Independently processes single images. Thus, even stationary objects can be detected. Can be used with moving or fixed cameras	- Adequate data and time are required to train CNN

**Table 2:** Proposed CNN architecture used in our research.

Layer Type	Number of Filters	Size of Feature Map	Size of Kernel	Number of Stride	Number of Padding
Image input layer		183 (height) × 119 (width) × 3 (channel)			
1st convolutional layer	96	87 × 55 × 96	11 × 11 × 3	2 × 2	0 × 0
ReLU layer		87 × 55 × 96			
Cross channel normalization layer		87 × 55 × 96			
Max pooling layer	1	43 × 27 × 96	3 × 3	2 × 2	0 × 0
2nd convolutional layer	128	43 × 27 × 128	5 × 5 × 96	1 × 1	2 × 2
ReLU layer		43 × 27 × 128			
Cross channel normalization layer		43 × 27 × 128			
Max pooling layer	1	21 × 13 × 128	3 × 3	2 × 2	0 × 0
3rd convolutional layer	256	21 × 13 × 256	3 × 3 × 128	1 × 1	1 × 1
ReLU layer		21 × 13 × 256			
4th convolutional layer	256	21 × 13 × 256	3 × 3 × 256	1 × 1	1 × 1
ReLU layer		21 × 13 × 256			
5th convolutional layer	128	21 × 13 × 128	3 × 3 × 256	1 × 1	1 × 1
ReLU layer		21 × 13 × 128			
Max pooling layer	1	10 × 6 × 128	3 × 3	2 × 2	0 × 0
1st fully connected layer		4096			
ReLU layer		4096			
2nd fully connected layer		1024			
ReLU layer		1024			
Dropout layer		1024			
3rd fully connected layer		2			
Softmax layer		2			
Classification layer (output layer)		2			

**Table 3:** Databases used in this research.

		DNHD-DB1	CVC-14 Database	KAIST Database
Number of images	Human	19,760	36,920	37,336
	Background	19,760	36,920	37,336
Number of channel		Color (3 channels)	Gray (1 channel)	Color (3 channels)
Width of human (background) image (min.~max.) (pixels)		15–219	64	21–106
Height of human (background) image (min.~max.) (pixels)		45–313	128	27–293
Environment of database collection		- Image acquisition using a static camera in surveillance environment - The height of the camera from the ground was about 6–10 m - Database was collected at various places (at 8–10 pm)	- Image acquisition using a camera mounted on the roof of a car while driving	

**Table 4:** Confusion matrix of recognition accuracies with original images by four-fold cross validation: (**a**–**d**) CVC-14 database; (**e**–**h**) DNHD-DB1; and (**i**–**l**) KAIST database (unit: %).

(**a**)			
**1st fold**		**Recognized**	
		**Human**	**Background**
Actual	Human	96.59	3.41
	Background	0.27	99.73
(**b**)			
**2nd fold**		**Recognized**	
		**Human**	**Background**
Actual	Human	97.11	2.89
	Background	0.22	99.78
(**c**)			
**3rd fold**		**Recognized**	
		**Human**	**Background**
Actual	Human	96.27	3.73
	Background	0.38	99.62
(**d**)			
**4th fold**		**Recognized**	
		**Human**	**Background**
Actual	Human	97.27	2.73
	Background	0.21	99.79
(**e**)			
**1st fold**		**Recognized**	
		**Human**	**Background**
Actual	Human	96.36	3.64
	Background	2.61	97.39
(**f**)			
**2nd fold**		**Recognized**	
		**Human**	**Background**
Actual	Human	95.99	4.01
	Background	7.01	92.99
(**g**)			
**3rd fold**		**Recognized**	
		**Human**	**Background**
Actual	Human	96.82	3.18
	Background	4.54	95.46
(**h**)			
**4th fold**		**Recognized**	
		**Human**	**Background**
Actual	Human	96.68	3.32
	Background	2.90	97.10
(**i**)			
**1st fold**		**Recognized**	
		**Human**	**Background**
Actual	Human	92.63	7.37
	Background	0.14	99.86
(**j**)			
**2nd fold**		**Recognized**	
		**Human**	**Background**
Actual	Human	83.02	16.98
	Background	0.32	99.68
(**k**)			
**3rd fold**		**Recognized**	
		**Human**	**Background**
Actual	Human	86.16	13.84
	Background	0.50	99.50
(**l**)			
**4th fold**		**Recognized**	
		**Human**	**Background**
Actual	Human	95.02	4.98
	Background	0.25	99.75

**Table 5:** Confusion matrix of recognition accuracies with HE processed images by four-fold cross validation: (**a**–**d**) CVC-14 database; (**e**–**h**) DNHD-DB1; and (**i**–**l**) KAIST database (unit: %).

(**a**)			
**1st fold**		**Recognized**	
		**Human**	**Background**
Actual	Human	96.08	3.92
	Background	0.26	99.74
(**b**)			
**2nd fold**		**Recognized**	
		**Human**	**Background**
Actual	Human	98.88	1.12
	Background	0.31	99.69
(**c**)			
**3rd fold**		**Recognized**	
		**Human**	**Background**
Actual	Human	95.99	4.01
	Background	0.46	99.54
(**d**)			
**4th fold**		**Recognized**	
		**Human**	**Background**
Actual	Human	96.42	3.58
	Background	0.31	99.69
(**e**)			
**1st fold**		**Recognized**	
		**Human**	**Background**
Actual	Human	92.55	7.45
	Background	3.73	96.27
(**f**)			
**2nd fold**		**Recognized**	
		**Human**	**Background**
Actual	Human	97.69	2.31
	Background	4.80	95.20
(**g**)			
**3rd fold**		**Recognized**	
		**Human**	**Background**
Actual	Human	97.17	2.83
	Background	6.34	93.66
(**h**)			
**4th fold**		**Recognized**	
		**Human**	**Background**
Actual	Human	97.15	2.85
	Background	3.16	96.84
(**i**)			
**1st fold**		**Recognized**	
		**Human**	**Background**
Actual	Human	93.03	6.97
	Background	0.14	99.86
(**j**)			
**2nd fold**		**Recognized**	
		**Human**	**Background**
Actual	Human	96.19	3.81
	Background	0.11	99.89
(**k**)			
**3rd fold**		**Recognized**	
		**Human**	**Background**
Actual	Human	97.98	2.02
	Background	0.13	99.87
(**l**)			
**4th fold**		**Recognized**	
		**Human**	**Background**
Actual	Human	96.50	3.50
	Background	0.14	99.86

**Table 6:** Average testing accuracies of human and background detection with original images (unit: %).

Database	PPV	TPR	ACC	F_Score
CVC-14 database	99.72	96.81	98.27	98.24
DNHD-DB1	95.73	96.46	96.09	96.09
KAIST database	99.64	89.21	94.60	94.07
Average	98.36	94.16	96.32	96.13

**Table 7:** Average testing accuracies of human and background detection with images processed by HE (unit: %).

Database	PPV	TPR	ACC	F_Score
CVC-14 database	99.65	96.84	98.25	98.23
DNHD-DB1	95.48	96.14	95.81	95.79
KAIST database	99.85	95.93	97.95	97.84
Average	98.33	96.30	97.34	97.29

**Table 8:** Average testing accuracies of human and background detection with combined database (unit: %).

Kinds of Input Image to CNN	PPV	TPR	ACC	F_Score
Original	99.31	93.44	96.44	96.26
HE	99.11	97.65	98.41	98.38

**Table 9:** Comparisons of average testing accuracies with previous and proposed methods with combined database (unit: %).

Methods	PPV	TPR	ACC	F_Score
HOG-SVM [16,42]	96.56	98.40	97.48	97.47
Proposed method	99.11	97.65	98.41	98.38
